# Supplementary material for: Integrated Transcriptome and Proteome Analysis Revealed the Regulatory Mechanism of Hypocotyl Elongation in Pakchoi
Source: Int J Mol Sci. 2023 Sep 7;24(18):13808. doi: 10.3390/ijms241813808 (PMC10531338; doi:10.3390/ijms241813808)
Supplement: Supplementary file 1 [file ijms-24-13808-s001.zip › ijms-2582609-supplementary.pdf]

Supplementary data

Table S1. Top 20 KEGG pathways of DEGs

Pathway	Ko _ ID	DEGs number	%
Plant-pathogen interaction	ko04626	152	9.07
Plant hormone signal transduction	ko04075	143	8.53
Carbon metabolism	ko01200	112	6.68
Starch and sucrose metabolism	ko00500	80	4.77
Photosynthesis	ko00195	80	4.77
Phenylpropanoid biosynthesis	ko00940	76	4.53
Biosynthesis of amino acids	ko01230	69	4.12
Carbon fixation in photosynthetic organisms	ko00710	60	3.58
Glyoxylate and dicarboxylate metabolism	ko00630	58	3.46
Protein processing in endoplasmic reticulum	ko04141	58	3.46
Ribosome	ko03010	55	3.28
Pentose and glucuronate interconversions	ko00040	47	2.80
Glycolysis / Gluconeogenesis	ko00010	43	2.57
Amino sugar and nucleotide sugar metabolism	ko00520	42	2.51
Endocytosis	ko04144	41	2.45
Spliceosome	ko03040	41	2.45
RNA transport	ko03013	39	2.33
ABC transporters	ko02010	39	2.33
Glycerolipid metabolism	ko00561	38	2.27
Cysteine and methionine metabolism	ko00270	35	2.09

Table S2. The expression of 6 DEGs in RNA-seq

Gene ID	FPKM (WT)	FPKM	FDR	FC	Regulated
BraC01g000200	21.0856	3.9336	3.7340E-09	-	down
BraC01g040730	6.1388	0.2226	2.8372E-20	-	down
BraC01g032200	0.1782	0.8921	1.3800E-05	1.9673	up
BraC02g002040	0.6190	14.4296	5.3766E-48	4.2434	up
BraC05g045610	0.2988	1.5292	1.7938E-06	2.0398	up
BraC06g009990	10.5523	0.2093	4.7462E-31	-	down

Table S3. qRT-PCR primer sequence

Genes ID	Primer discription	Primer sequence (5'-3')
BraC02g002040	Forward	GTTGTTCATGAGTATCTGGTGG
	Reverse	AAGTATTCATGTCGCCTCTCC
BraC01g032200	Forward	TATCCATGGAAGTAGAGTTTCTGG
	Reverse	AAAGCTGTAGACATCAGAGGG
BraC05g045610	Forward	GTATAGTGTCAGAGTTCCTTCCAC
	Reverse	GTCAGCTACCTTAACAGTCCA
BraC01g000200	Forward	GACGGAGAACACGACATTGAC
	Reverse	TCCCGACAAGAACATGATTCC
BraC01g040730	Forward	CTCTTCAGAGCAAGTTCTTTGAG
	Reverse	GACCAAGCTTAGGCTTCTCC
BraC06g009990	Forward	TGAAGGATTAAGAGACATGAGGG
	Reverse	GAGATACGACTCGTACACCT
GAPDH	Forward	GACTGGAGAGGTGGAAGAGCC
	Reverse	ACTGAAACATCAACGGTGGGA

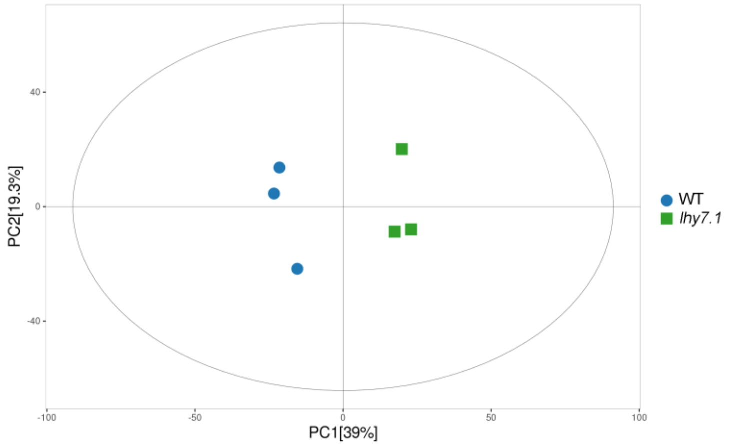

Figure S1. PCA of proteome
